# Supplementary material for: Development of a Quadruplex RT-qPCR for the Detection of Porcine Astrovirus, Porcine Sapovirus, Porcine Norovirus, and Porcine Rotavirus A
Source: Pathogens. 2024 Nov 29;13(12):1052. doi: 10.3390/pathogens13121052 (PMC11728830; doi:10.3390/pathogens13121052)
Supplement: Supplementary file 1 [file pathogens-13-01052-s001.zip › pathogens-3304482-supplementary.pdf]

Supplementary Materials

Table S1 The information of PoAstV

Strain	Accession No.	Origin	Isolation Date	Upload Date	Genotype
275R-116493	MW784096.1	China	/	2-Aug-2022	AstV1
138bR-178666	MW784095.1	China	/	2-Aug-2022	AstV1
HUN	GU562296.1	Hungary	2007	6-Jan-2011	AstV2
Por-411	MG003352.1	India	2016	10-Oct-2019	AstV3
Por-408	MG003351.1	India	2016	10-Oct-2019	AstV3
Por-403	MG003350.1	India	2016	10-Oct-2019	AstV3
Por-393	MG003348.1	India	2016	10-Oct-2019	AstV3
Por-392	MG003347.1	India	2016	10-Oct-2019	AstV3
Por-391	MG003346.1	India	2016	10-Oct-2019	AstV3
Por-386	MG003345.1	India	2016	10-Oct-2019	AstV3
Dali-5	KX431950.1	China	28-Sep-2015	23-Apr-2017	AstV3
Dali-4	KX431949.1	China	28-Sep-2015	23-Apr-2017	AstV3
Dali-3	KX431948.1	China	28-Sep-2015	23-Apr-2017	AstV3
Dali-2	KX431947.1	China	28-Sep-2015	23-Apr-2017	AstV3
Dali-1	KX431946.1	China	28-Sep-2015	23-Apr-2017	AstV3
Por-135	KT757532.1	India	2013	1-Sep-2016	AstV3
Por-30	KT757529.1	India	2013	1-Sep-2016	AstV3
IVRI/24	KM083103.1	India	05-Apr-2014	15-Jul-2016	AstV3
PAstK-126	JQ696852.1	South Korea	2011	4-Apr-2013	AstV3
PAstK-124	JQ696851.1	South Korea	2011	4-Apr-2013	AstV3
PAstK-123	JQ696850.1	South Korea	2011	4-Apr-2013	AstV3
PAstK-114	JQ696847.1	South Korea	2011	4-Apr-2013	AstV3
PAstK-103	JQ696845.1	South Korea	2011	4-Apr-2013	AstV3
PAstK-73	JQ696843.1	South Korea	2011	4-Apr-2013	AstV3
PAstK-54	JQ696839.1	South Korea	2011	4-Apr-2013	AstV3
/	OM104029.1	South Africa	Mar-2021	26-Jul-2024	AstV3
PAstK-12	JQ696833.1	South Korea	2011	4-Apr-2013	AstV3
AH4-2	OL695849.1	China	2021	14-Nov-2022	AstV3
SP-VT41	ON792972.1	Spain	16-May-2018	26-Oct-2022	AstV4
SP-VC9	ON792970.1	Spain	25-Jan-2017	26-Oct-2022	AstV4
SP-VT9	ON792969.1	Spain	31-Jan-2018	26-Oct-2022	AstV4
SP-VC41	ON792968.1	Spain	19-Oct-2017	26-Oct-2022	AstV4
SP-VC29	ON792967.1	Spain	06-Jun-2017	26-Oct-2022	AstV4
HgTa2-1-2	LC201607.1	Japan	2015	16-May-2017	AstV4
286-165764	MW784090.1	China	/	2-Aug-2022	AstV5

Table S2 The information of PoSaV

Strain	Accession No.	Origin	Isolation Date	Upload Date	Genotype
IA27912-A	MK965902.1	USA	2018	13-Jan-2020	GIII
KS8.7-A	MK965899.1	USA	2019	13-Jan-2020	GIII
NE7.5	MK965900.1	USA	2019	13-Jan-2020	GIII
P361A-2	MK962339.1	Spain	04-Jul-2017	17-Nov-2019	GIII
P452	MK962338.1	Spain	22-Sep-2017	17-Nov-2019	GIII
HLJ01	MK378994.1	China	Apr-2017	25-Jun-2019	GIII
1ah	KX688105.1	China	2010	31-Aug-2018	GIII
HgTa3-1	LC215884.1	Japan	Dec-2016	27-Sep-2017	GIII
HgYa2-1	LC215881.1	Japan	Dec-2016	27-Sep-2017	GIII
LL14	KT945133.1	USA	27-Mar-2002	9-Jan-2016	GIII
JJ259	KT922089.1	USA	/	9-Jan-2016	GIII
TCA-Cowden	KT922088.1	USA	20-May-2010	9-Jan-2016	GIII
Cowden	KT922087.1	USA	15-Nov-1979	9-Jan-2016	GIII
IA_0365	MW316759.1	USA	01-Oct-2019	10-May-2021	GIII
NE_7211-4	MW316755.1	USA	18-Jun-2019	10-May-2021	GIII
NE_7211-3	MW316754.1	USA	18-Jun-2019	10-May-2021	GIII
MI_64533	MW316749.1	USA	01-Sep-2020	10-May-2021	GIII
IL_16624	MW316747.1	USA	17-Mar-2020	10-May-2021	GIII
IN_17168-3A	MW316746.1	USA	10-Mar-2020	10-May-2021	GIII
IN_17168-1	MW316745.1	USA	10-Mar-2020	10-May-2021	GIII
HW20	HM346629.1	South Korea	2007	25-Jul-2016	GIII
GD122	EU381222.1	China	2007	26-Jul-2016	GIII
LL14	AY425671.1	USA	2003	9-Jun-2004	GIII
NC-QW270	AY826426.1	USA	2005	26-Jul-2016	GIII

Table S3 The information of PoNoV

Strain	Accession No.	Origin	Isolation Date	Upload Date	Genotype
sw59	GQ149615.1	China	Feb-2009	25-Jul-2016	GII.19
Ch6	HQ392821.1	China	Aug-2009	1-Jun-2012	GII.19
sw86	JN644280.1	Netherlands	2009	1-Aug-2013	GII.19
Vet14-S08257	FJ843084.1	Denmark	/	15-Apr-2009	GII.19
Vet53-S08140	FJ843087.1	Denmark	/	15-Apr-2009	GII.19
sw42	GQ149616.1	China	Feb-2009	25-Jul-2016	GII.19
90186-2	MN605620.1	Italy	26-Oct-2018	1-May-2020	GII.11
90186-1	MN605619.1	Italy	26-Oct-2018	1-May-2020	GII.11
90275-3	MN605629.1	Italy	24-Jan-2019	1-May-2020	GII.11
90275-2	MN605628.1	Italy	24-Jan-2019	1-May-2020	GII.11
90233-1	MN605626.1	Italy	06-Dec-2018	1-May-2020	GII.11
90114-5	MN605616.1	Italy	06-Feb-2018	1-May-2020	GII.11
90114-3	MN605615.1	Italy	06-Feb-2018	1-May-2020	GII.11
90114-2	MN605614.1	Italy	06-Feb-2018	1-May-2020	GII.11
90274-2	MN605612.1	Italy	24-Jan-2019	1-May-2020	GII.11
90274-1	MN605611.1	Italy	24-Jan-2019	1-May-2020	GII.11
17diapd90019	MN567956.1	Italy	2017	1-May-2020	GII.11

Table S4 The information of PoRVA

Strain	Accession No.	Origin	Isolation Date	Upload Date	Genotype
SCMY	MH320796.1	China	Jan-2018	12-Feb-2019	I5
F37	MH238292.1	Spain	01-Mar-2017	6-Feb-2019	I5
K71	MF940442.1	South Korea	2006	25-Jul-2018	I5
K72	MF940439.1	South Korea	2006	25-Jul-2018	I5
HN03	MH021179.1	China	Dec-2015	1-Apr-2018	I5
SCQL-5-2	MG029105.1	China	May-2017	25-Mar-2018	I5
SCQL-5-1	MG029103.1	China	May-2017	25-Mar-2018	I5
YT	OR232953.1	China	2022	20-Sep-2023	I5
14150_54	KX363349.1	Vietnam	12-Mar-2012	2-Jul-2016	I5
14150_53	KX363332.1	Vietnam	12-Mar-2012	2-Jul-2016	I5
KY-2022	OR127198.1	China	01-Oct-2022	30-Aug-2023	I5
SXXA	OR091161.1	China	2020	30-Aug-2023	I5
HB-TS96	OM735819.1	China	2021	14-Dec-2022	I5
FX17	OM362095.1	China	2021	7-Dec-2022	I5
LS00006_OSU	KR052760.1	USA	12-Apr-1975	28-Apr-2015	I5
MRC-DPRU1568	KP753194.1	South Africa	2008	1-Apr-2015	I5
DPRU1487	KP753126.1	South Africa	2007	25-Sep-2015	I5
GDJM1	OP718289.1	China	2022	3-Dec-2022	I5
GXGG	OL884613.1	China	Aug-2017	15-Nov-2022	I5
S20-0073	OM982795.1	Switzerland	2020	12-Jun-2022	I5
S20-0074	OM982794.1	Switzerland	2020	12-Jun-2022	I5
OSU-C5111	KJ450847.1	Spain	2010	14-Jun-2014	I5
MRC-DPRU1567	KJ752489.1	South Africa	Mar-2008	13-May-2014	I5
C-1	KF500222.1	South Korea	2006	15-Jan-2014	I5
174-1	KF500211.1	South Korea	2006	24-Jul-2014	I5
Jan-42	KF500189.1	South Korea	2006	15-Jan-2014	I5
K5	JX971573.1	South Korea	2004	27-Jan-2013	I5
DN30209	JN977137.1	China	01-Oct-2009	13-May-2012	I5

Supplementary Materials

A: PoAstV

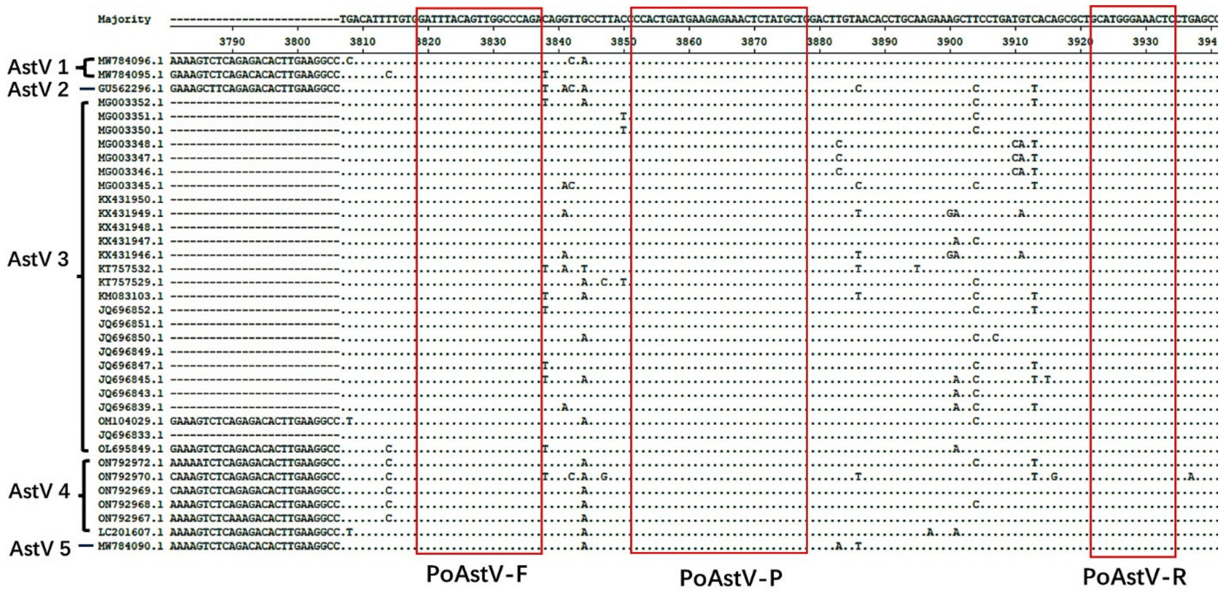

B: PoSaV

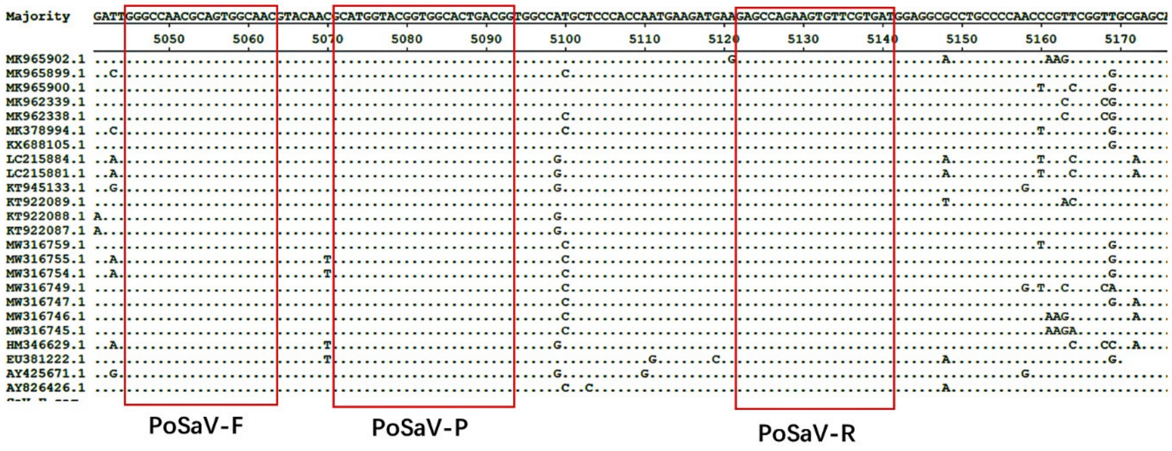

C: PoNoV

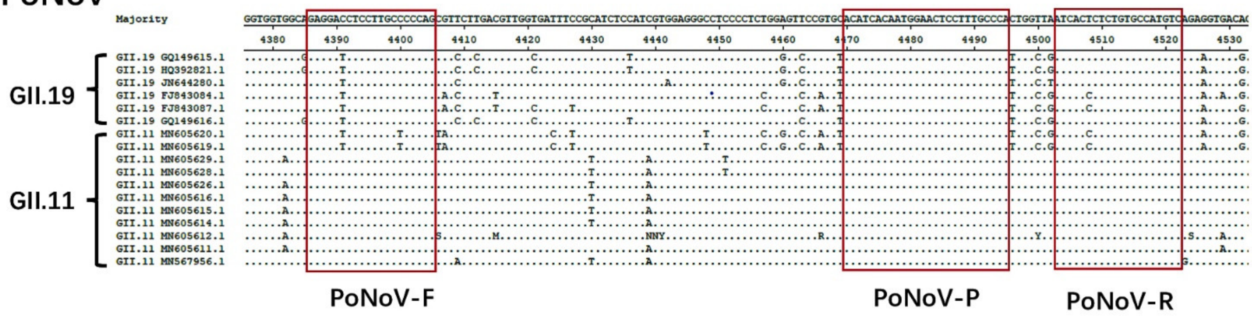

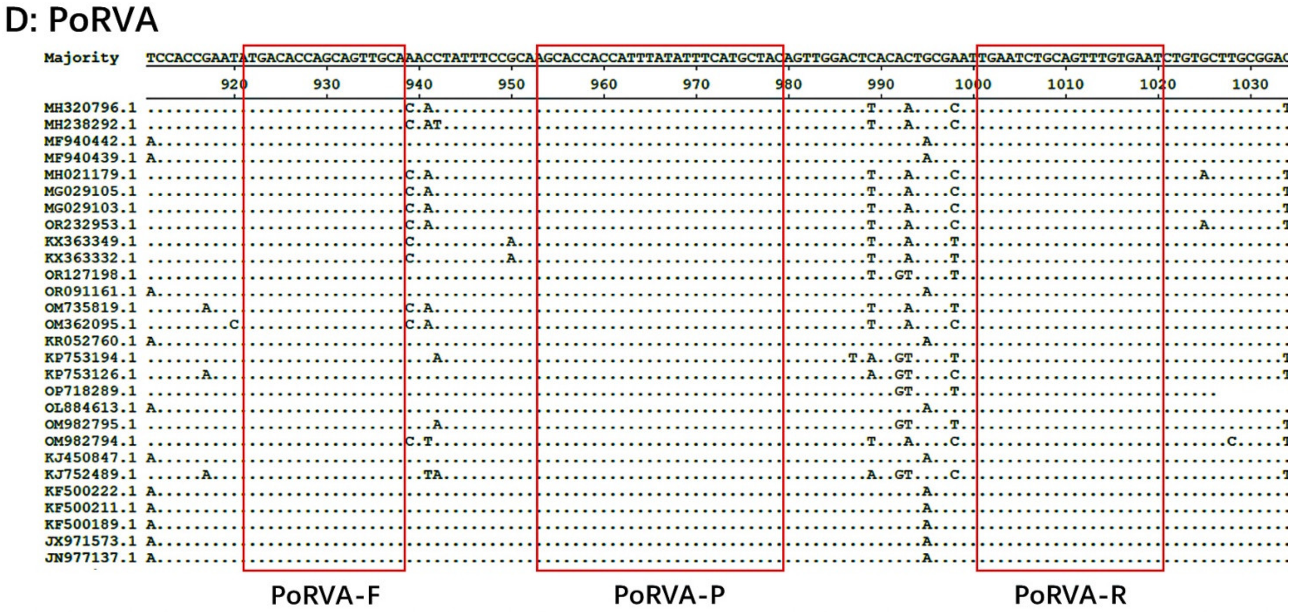

Figure S1. The multiple sequence alignments of PoAstV (A), PoSaV (B), PoNoV (C), and PoRVA (D). The locations of Forward (F)/Reverse (R) primers, and Probe (P) are shown.
